# The Narrow QRS Deception: Alternating Bundle Branch Block Uncovered on Ambulatory Monitoring After TAVR

**DOI:** 10.1016/j.jaccas.2026.107734

**Published:** 2026-04-22

**Authors:** Serhat Kesriklioglu, Ahmet Taha Sahin, Yakup Alsancak, Ahmet Lutfu Sertdemir, Enes Elvin Gul

**Affiliations:** Department of Cardiology, School of Medicine, Necmettin Erbakan University, Konya, Turkey

**Keywords:** bundle branch block, conduction system, transcatheter aortic valve replacement

## Abstract

**Background:**

Conduction disturbances are common after transcatheter aortic valve replacement (TAVR), whereas alternating bundle branch block reflects advanced conduction system disease.

**Case Summary:**

A 76-year-old man underwent TAVR with an initially normal postprocedural electrocardiogram (ECG). Six days later, he presented with syncope. The admission ECG demonstrated wide QRS complexes. A retrospective review of early 12-lead Holter monitoring revealed alternating bundle branch block. Conduction system pacing was performed.

**Discussion:**

This case illustrates how dynamic conduction instability after TAVR may remain undetected on a resting ECG and require extended rhythm assessment for early recognition.

**Take-Home Messages:**

Extended rhythm surveillance after TAVR can detect transient or intermittent conduction abnormalities not apparent on a standard ECG. Early identification of such changes may enable timely electrophysiological evaluation and appropriate device therapy.

## Visual Summary

**Figure:**
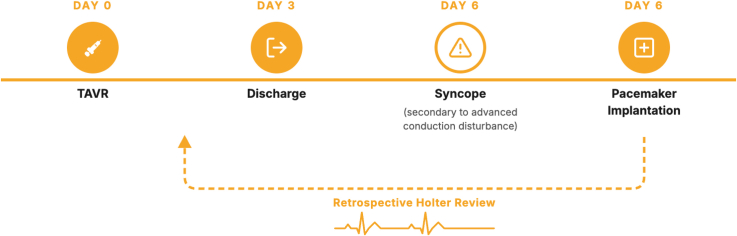

Conduction disturbances are common after transcatheter aortic valve replacement (TAVR), with new-onset left bundle branch block (LBBB) being the most frequently observed abnormality.^1^ In contrast, alternating bundle branch block (BBB) is rare and signifies advanced, bilateral His-Purkinje system involvement that may precede high-grade atrioventricular block.^2^ Early identification of dynamic or intermittent post-TAVR conduction abnormalities remains challenging because standard 12-lead electrocardiography (ECG) often fails to capture transient changes. Twelve-lead Holter monitoring may provide unique diagnostic insights into evolving conduction system disease. We describe a case of post-TAVR alternating BBB detected only through Holter monitoring, ultimately guiding the device therapy.

**Take-Home Messages**

• Alternating bundle branch block after TAVR reflects progressive conduction system injury with a high risk for advanced bradyarrhythmias and tachyarrhythmias.
• Extended 12-lead Holter monitoring is superior to resting electrocardiograms for detecting dynamic post-TAVR conduction disturbances, enabling timely electrophysiological evaluation and appropriate device therapy.

## Case Presentation

**Figure 1:**
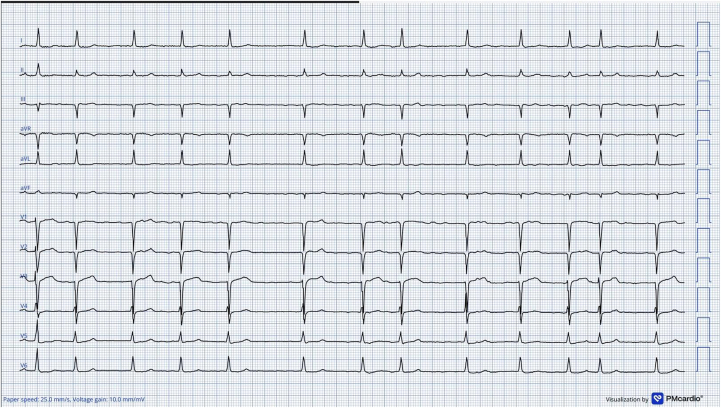
Postprocedural 12-Lead Electrocardiogram Demonstrating Atrial Fibrillation With a Narrow QRS Complex and Normal Ventricular Response (Electrocardiogram-Generated Using the AI-Enabled PMcardio Platform)

A 76-year-old man with ischemic cardiomyopathy, permanent atrial fibrillation (AF), and chronic coronary syndrome underwent TAVR for symptomatic severe aortic stenosis with successful deployment of a Myval transcatheter aortic bioprosthesis (Meril Life Sciences). Preprocedural multidetector computed tomography demonstrated an annulus perimeter-derived diameter of 25.8 mm (area-derived diameter = 25.1 mm), a left ventricular outflow tract diameter of 25 mm, sinus of Valsalva diameters of 29.8/27.7/30.2 mm, a sinotubular junction diameter of 24.1 mm, and coronary heights of 18.6 mm (right coronary artery) and 14.3 mm (left coronary artery), with minimal calcification. Based on these findings, a 26-mm Myval valve was selected. The immediate postprocedural ECG showed AF with a narrow QRS complex and no new conduction abnormality (Figure 1). As part of an institutional research protocol, a 48-hour 12-lead Holter monitor was used postoperatively; however, the patient was discharged early at his request, and the recordings were not reviewed before discharge.

**Figure 2:**
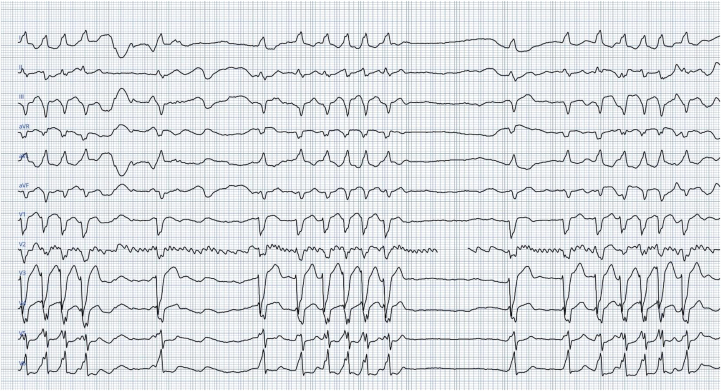
Electrocardiogram Recorded on Postoperative Day 6 Demonstrating Atrial Fibrillation With a Wide QRS Complex of Left Bundle Branch Block, Tachy-Brady Episodes, and Pauses (Electrocardiogram-Generated Using the AI-Enabled PMcardio Platform)

Six days later, he presented to the emergency department with syncope and hypotension. The admission ECG revealed AF with a wide QRS complex compatible with LBBB, tachy-brady episodes, and pauses (Figure 2). The patient was immediately admitted to the intensive care unit, and a decision to implant a permanent pacemaker was made. Because of impaired systolic function (left ventricular ejection fraction = 30%), we planned to proceed with cardiac resynchronization therapy-defibrillator implantation.

**Figure 3:**
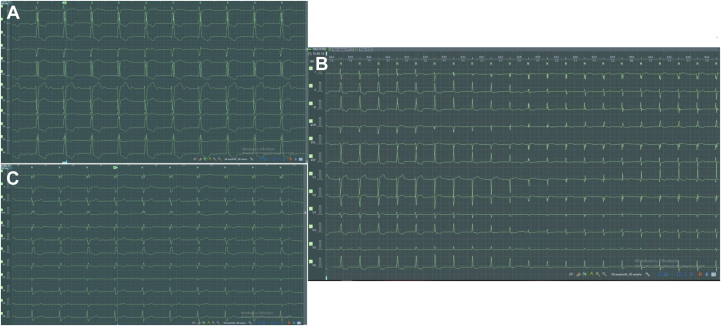
12-Lead ECG Holter Recordings (A) Holter tracing from the early post–transcatheter aortic valve replacement period demonstrating a junctional rhythm with a wide QRS complex (QRSd: 128 milliseconds) and an LBBB morphology (recorded at 50 mm/s). (B) Holter recording showing sequential transitions from LBBB to a narrow QRS complex and subsequently to RBBB, consistent with an alternating bundle branch block pattern without a significant change in heart rate. (C) Holter recording demonstrating a subsequent complete RBBB morphology (QRSd: 128 milliseconds) (recorded at 50 mm/s). LBBB = left bundle branch block; RBBB = right bundle branch block.

**Figure 4:**
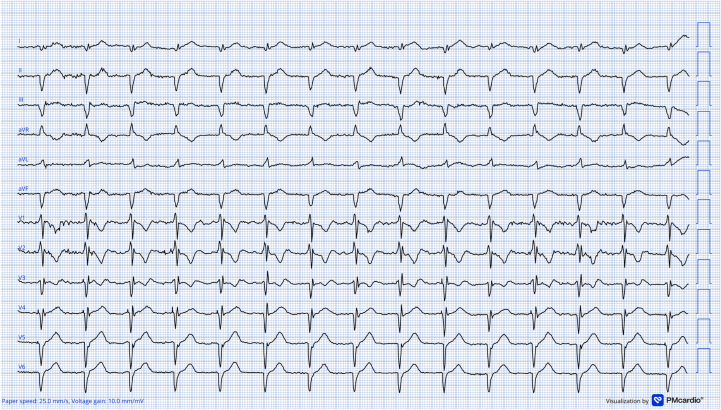
Postimplantation 12-Lead Electrocardiogram Demonstrating a Stable Paced Rhythm With a Narrow QRS Morphology (QRS Duration = 127 milliseconds) After Left Bundle Branch Area Pacing–Optimized Cardiac Resynchronization Therapy

A retrospective review of the post-TAVR Holter study showed sequential transitions from LBBB to a narrow QRS complex, then to incomplete right bundle branch block (RBBB), and ultimately to complete RBBB (Figure 3), consistent with alternating BBB and rapidly progressive conduction system disease. The patient subsequently underwent left bundle branch area pacing–optimized cardiac resynchronization therapy, after which he remained clinically stable and was discharged in good condition (Figure 4).

## Discussion

Conduction disturbances are among the most frequent electrical complications after TAVR, with new-onset LBBB occurring in up to one-third of patients.^3^ In contrast, alternating BBB is rare and reflects bilateral His-Purkinje system involvement, often preceding high-grade atrioventricular block. Alternating QRS morphologies, especially transitions between LBBB- and RBBB-like patterns, indicate marked conduction system vulnerability and should trigger concern for imminent advanced block.^4^^,^^5^ Kulkarni et al^6^ emphasized that alternating BBB results from variable conduction delay in both bundle branches rather than fixed block, and its recognition carries important prognostic implications. Such findings highlight the need for careful postprocedural monitoring, even in patients initially presenting with narrow QRS complexes. Our case reinforces these observations, demonstrating how early conduction instability may evolve rapidly and unpredictably after valve implantation.

A unique feature of this case was the extended 12-lead Holter recording, which revealed sequential QRS transitions from an LBBB-like morphology to a narrow QRS complex, then to incomplete RBBB, and finally to complete RBBB, all within the same recording period. The preservation of heart rate during these transitions suggests that the changes were not rate dependent but rather reflected fluctuating conduction delay in both bundle branches. This dynamic alternation likely represents progressive mechanical compression, inflammatory edema, or transient ischemia affecting the left and right bundle branches after TAVR deployment. The initial junctional rhythm observed on a Holter recording may also indicate transient injury at the atrioventricular nodal or Hisian level, consistent with prior findings showing that post-TAVR junctional rhythm is associated with a high risk of subsequent pacemaker implantation.^7^^,^^8^ These Holter-detected changes provided critical insights into the evolving conduction instability that was not apparent on a standard resting ECG.

Although RBBB after TAVR is less common than new-onset LBBB, its presence—especially when accompanied by fascicular involvement or alternating QRS patterns—should heighten suspicion for diffuse, multilevel conduction system injury. The combination of RBBB and left septal fascicular block has previously been shown to represent trifascicular involvement and to markedly increase the risk of complete atrioventricular block and sudden cardiac death.^9^ Our patient's evolution from incomplete to complete RBBB following a preceding LBBB-like pattern is consistent with this concept of bilateral and progressive conduction system compromise. These findings underscore that non-LBBB conduction disturbances should not be regarded as benign and require equally monitoring. The dynamic nature of these changes further illustrates how conduction system injury after TAVR can evolve rapidly, even in patients with initially stable postprocedural ECGs. In such scenarios, early recognition is essential for timely intervention.

Recent expert consensus reports and studies emphasize a structured, risk-based approach to managing post-TAVR conduction disturbances, incorporating serial ECG evaluation, telemetry, and selective ambulatory rhythm monitoring rather than relying solely on resting ECGs.^10^^,^^11^ Rodés-Cabau et al^12^ particularly underscored the value of standardized pathways integrating continuous rhythm assessment and electrophysiological testing to identify patients at risk for delayed high-grade atrioventricular block before overt clinical deterioration occurs. The presence of conduction abnormalities such as LBBB or RBBB post-TAVR may identify a group of patients who would benefit from extended ambulatory ECG monitoring. However, in patients with no ECG changes after the procedure, the risk for late conduction disturbances is lower compared with patients with preexistent RBBB or post-TAVR LBBB (1.2% vs 13.2% and 8.5%, respectively).^11^ According to these recommendations, our patient, who initially had narrow QRS AF and no new conduction abnormality after TAVR, would not have met conventional criteria for early device evaluation. However, extended 12-lead Holter monitoring revealed alternating bundle branch conduction and rapid progression of conduction system injury that were entirely absent on standard surveillance. This case therefore illustrates how Holter-based rhythm assessment, although not routinely mandated for patients with no ECG changes in current algorithms, may provide critical incremental diagnostic information and help refine early pacing decisions in selected post-TAVR patients.

## Conclusions

This case illustrates that alternating BBB may serve as an early marker of progressive His-Purkinje system injury, detectable only through extended rhythm monitoring even in patients with no ECG changes after TAVR. Timely identification of such dynamic conduction changes can guide earlier electrophysiological evaluation and individualized pacing strategies, helping to prevent potentially life-threatening cardiac events.

**Visual Summary:**
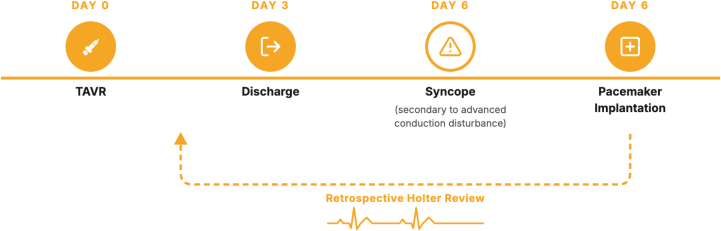
Timeline of the Case TAVR = transcatheter aortic valve replacement.

## Funding Support and Author Disclosures

The authors have reported that they have no relationships relevant to the contents of this paper to disclose.
